# Variation in water contact behaviour and risk of *Schistosoma mansoni* (re)infection among Ugandan school-aged children in an area with persistent high endemicity

**DOI:** 10.1186/s13071-021-05121-6

**Published:** 2022-01-06

**Authors:** Suzan C. M. Trienekens, Christina L. Faust, Fred Besigye, Lucy Pickering, Edridah M. Tukahebwa, Janet Seeley, Poppy H. L. Lamberton

**Affiliations:** 1 Institute of Biodiversity, Animal Health and Comparative Medicine, College of Medical, Veterinary and Life Sciences, University of Glasgow, Glasgow, UK; 2 Wellcome Centre for Integrative Parasitology, College of Medical, Veterinary and Life Sciences, University of Glasgow, Glasgow, UK; 3 Institute of Health & Wellbeing, College of Social Sciences, University of Glasgow, Glasgow, UK; 4 Vector Control Division, Ministry of Health, Kampala, Uganda; 5 Medical Research Council/Uganda Virus Research Institute, Entebbe, Uganda; 6 Department of Global Health and Development, Faculty of Public Health and Policy, London School of Hygiene and Tropical Medicine, London, UK

**Keywords:** Schistosomiasis, *Biomphalaria*, Infection intensity, Exposure, Ethnography, Snail survey, Vector control, Water-borne, Transmission, Seasonality

## Abstract

**Background:**

Annual mass drug administration with praziquantel has reduced schistosomiasis transmission in some highly endemic areas, but areas with persistent high endemicity have been identified across sub-Saharan Africa, including Uganda. In these areas many children are rapidly reinfected post treatment, while some children remain uninfected or have low-intensity infections. The aim of this mixed-methods study was to better understand variation in water contact locations, behaviours and infection risk in school-aged children within an area with persistent high endemicity to inform additional control efforts.

**Methods:**

Data were collected in Bugoto, Mayuge District, Uganda. Two risk groups were identified from a longitudinal cohort, and eight children with no/low-intensity infections and eight children with reinfections were recruited. Individual structured day-long observations with a focus on water contact were conducted over two periods in 2018. In all identified water contact sites, four snail surveys were conducted quarterly over 1 year. All observed *Biomphalaria* snails were collected, counted and monitored in the laboratory for *Schistosoma mansoni* cercarial shedding for 3 weeks.

**Results:**

Children came into contact with water for a range of purposes, either directly at the water sources or by coming into contact with water collected previously. Although some water contact practices were similar between the risk groups, only children with reinfection were observed fetching water for commercial purposes and swimming in water sources; this latter group of children also came into contact with water at a larger variety and number of sites compared to children with no/low-intensity infection. Households with children with no/low-intensity infections collected rainwater more often. Water contact was observed at 10 sites throughout the study, and a total of 9457 *Biomphalaria* snails were collected from these sites over four sampling periods. Four lake sites had a significantly higher *Biomphalaria choanomphala* abundance, and reinfected children came into contact with water at these sites more often than children with no/low-intensity infections. While only six snails shed cercariae, four were from sites only contacted by reinfected children.

**Conclusions:**

Children with reinfection have more high-risk water contact behaviours and accessed water sites with higher *B. choanomphala* abundance, demonstrating that specific water contact behaviours interact with environmental features to explain variation in risk within areas with persistent high endemicity. Targeted behaviour change, vector control and safe water supplies could reduce reinfection in school-aged children in these settings.

**Graphical Abstract:**

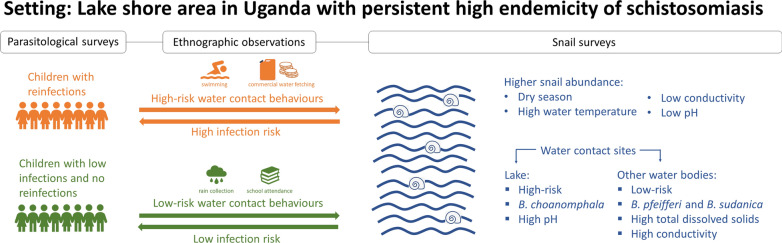

**Supplementary Information:**

The online version contains supplementary material available at 10.1186/s13071-021-05121-6.

## Background

Schistosomiasis is a neglected tropical disease caused by a water-borne parasitic infection. The World Health Organization (WHO) recently launched the 2021–2030 roadmap for neglected tropical diseases in which the global goal to eliminate schistosomiasis as a public health problem by 2030 is outlined (defined as < 1% of infections classified as high intensity) [1]. The disease disproportionately affects school-aged children (SAC) [2], and in areas where prevalence of schistosomiasis is  ≥ 50% in SAC, the WHO recommends community-wide annual mass drug administration (MDA) with praziquantel to kill adult worms and reduce egg production. The target is to treat > 75% of SAC and at-risk adults in these highly endemic areas to prevent morbidity from schistosomiasis and ultimately reduce transmission [3].

After years of MDA, areas with persistent high prevalence and high intensity of infection and associated high morbidity, often termed persistent hotspots, remain in sub-Saharan Africa [4]. In Uganda, where an estimated 29% of the population are SAC [5], the target coverage for MDA was reached in only 43% of endemic districts in 2019, with a reported 61% of SAC receiving MDA across the country [6]. Although treatment can be highly effective in reducing morbidity (primarily caused by the eggs), treatment does not prevent subsequent reinfection, which is a key challenge in areas with active transmission [7]. Even in settings where target coverage for SAC is almost reached [8], more than half of children can become reinfected 6 months after clearance of parasites with successful treatment, with the majority of reinfections detected only 9 weeks after treatment [10]. Understanding both the behaviours associated with exposure and the biological drivers of infection in these communities is crucial to achieving WHO global targets.

The lack of available and/or access to safe water is a main driver in perpetuating the burden of schistosomiasis [11]. Although three in four children living in rural areas in Uganda are reported to have access to safe drinking water [12], often multiple water sources, including unsafe water sources, are accessed for additional uses, such as for domestic, personal care and recreational purposes [13, 14]. If no protective measures or preventative treatment of water are used [15], contact with this water can expose children to *Schistosoma mansoni* parasites that develop in the intermediate hosts, species of *Biomphalaria* snails. Infection risk can increase by exposure factors, such as duration of water contact, frequency of water contact and level of submersion in water [16–18] and with snail presence and abundance [19]. Some environmental factors have been found to be associated with the abundance of *Biomphalaria* snails, such as dry seasons and little rainfall [20–22], as well as lake sites in comparison to inland habitats [23]. Studies on *Biomphalaria* abundance and physicochemical water factors have reported different findings, including associations with high pH [24], low pH [25, 26], low conductivity [24, 25], high temperature [23] and low turbidity [27, 28], whereas some studies did not find any associations at all [20, 29].

Some children in areas with persistent high endemicity remain uninfected or have such low-intensity infections that they are indetectable, suggesting variation in host susceptibility, biological exposure and/or behavioural susceptibility. While research has focussed on individuals who are persistently infected—either through rapid reinfection or because they are never treated with MDA [8]—there has been minimal focus on consistently uninfected children. Identifying the risk behaviours of this group of children and comparing their behaviour to children with high-intensity infections and/or rapid reinfections will provide important insights and enable recommendations to be made on locally feasible ways to reduce infections and reinfections among SAC and across the wider community. We reason that it might be easier for people to act on recommendations to reduce water contact when these recommendations are based on water contact behaviours and locations already known to be performed and used within specific communities.

In this mixed-methods study we identified groups of SAC who are rarely infected and who are rapidly reinfected, respectively, within an area with persistently high schistosomiasis endemicity. We used ethnographic observations, parasitological surveys and snail surveys to better understand variations in behaviour and exposure risk to improve guidance for sustainable integrated control.

The specific objectives of this study were: (i) to identify water contact risk behaviours and locations of water contact among SAC; (ii) to compare water contact behaviours between children with rapid reinfection and children with no or low-intensity infections; (iii) to assess variation in environmental infection risk at all water contact sites.

## Methods

### Study site

**Fig. 1:**
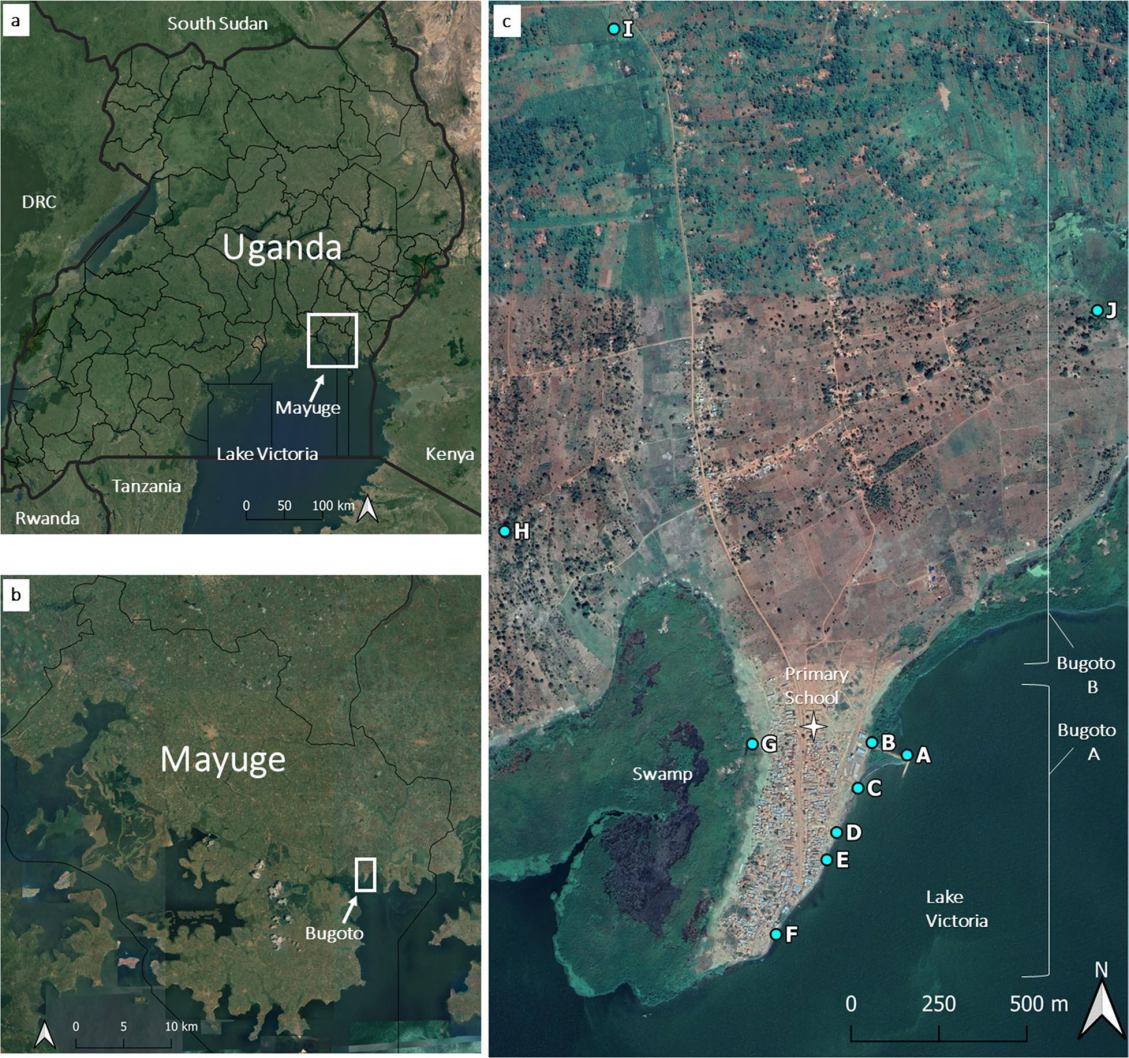
Map of study site. **a** Satellite imagery (source: Google Maps) showing the districts of Uganda (source: Uganda Bureau of Statistics, Kampala, Uganda). **b** Close-up of Mayuge District with the location of the study area indicated. **c** Map showing the villages of Bugoto A and B and their respective water contact sites (*A*–*J*); these sites were identified through ethnographic observations and are the sites where all snail surveys were carried out

All data for this study were collected in Bugoto, Mayuge District, south-eastern Uganda (Fig. 1a, b), a community situated on the shores of Lake Victoria. Persistent high prevalence of schistosomiasis has been reported in Bugoto [9, 30]. The community comprises two villages: Bugoto A (densely populated, close to the lake, predominately a fishing village) and Bugoto B (sparsely populated, inland from the lake, predominately a farming village) (Fig. 1c). Christianity and Islam are the main religions in the community, and the majority of people are of the Basoga tribal group. We selected participants from a primary school (Bugoto Lake View) that is situated between Bugoto A and B. It has students from both villages and is the main primary school in the area and the only public one.

### Cohort selection

**Fig. 2:**
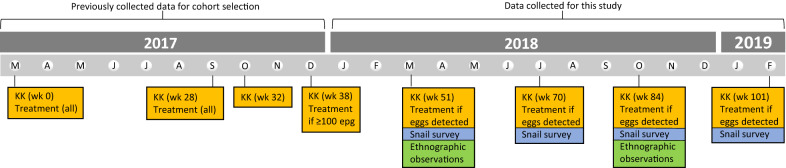
Timeline (starting in March 2017 (*M*)) of data collection for cohort selection, parasitological surveys (yellow boxes), snail surveys (blue boxes) and ethnographic observations (green boxes). Abbreviations: KK, kato-katz thick smears, taken in duplicate over 1 to 3 days

To better understand water contact behaviour and differences in exposure, two groups of children with differing *S. mansoni* parasitological statuses were selected among children from a larger longitudinal study cohort of SAC (SCHISTO_PERSIST) (*n* = 274; [31]). In this larger cohort, presence of *S. mansoni* eggs and mean infection intensities were calculated from 3 days of duplicate kato-katz thick smears at four time points: March 2017 (week 0), September 2017 (week 28), October 2017 (week 32) and December 2017 (week 38) (Fig. 2). After both the March and September sample collections, all children were treated with 40 mg/kg praziquantel, regardless of infection status. In December 2017, only children with ≥ 100 eggs per gram (epg) of stool were treated.

Children were selected and placed into two groups based on the results of this longitudinal parasitological survey. Eight children with rapid reinfection (CRI) were selected for one group; CRI were defined as those who were infected at weeks 0 and 28, and who although cleared infection following treatment (0 epg at week 32) were rapidly reinfected 9 weeks post treatment (week 38). An additional eight children with low-intensity infection (CLI) were chosen for the second group; CLI were defined as having no detectable or low-intensity infections (< 100 epg) at weeks 0 and 28, and who after successful clearance of infections in week 32 (0 epg) still had 0 epg at 9 weeks following treatment (week 38) (see Additional file 1: Table S1 for cohort specific parasitological data used for selection). We aimed for even representation of village residence, gender, age and both religions across the groups.

### Data collection

#### Parasitological surveys

We carried out additional parasitological surveys in 2018 and 2019 for the participating CRI and CLI present at weeks 51, 70, 82 and 101 after initial SCHISTO_PERSIST cohort selection (Fig. 2). Stool samples were collected on 1 to 3 consecutive days and examined by duplicate standard kato-katz thick smear method [32] to determine the presence of *S. mansoni* eggs and to quantify infection intensity. All children detected with egg presence were treated with 40 mg/kg praziquantel at each time point.

#### Ethnographic observations

Ethnographic methods for this study have been described in detail elsewhere [9]. In short, a team comprising one researcher and one community member/translator conducted two individual day-long ethnographic observations, for a total of 32 days for all selected children, of the participants’ daily activities at home, school and in the community, with a focus on water contact behaviours. The 2 days of observation for each child was split to incorporate differences between school days and weekends/holidays and different seasons (March [rainy] and October [light rainy] 2018; Fig. 2). GPS coordinates of locations where children were observed to contact a water body were recorded with a Garmin™ eTrex10 (Garmin Ltd., Olathe, KS, USA), and these data informed the sites for the snail surveys. Non-structured observation transect walks were performed throughout the data collection periods, across all days of the week, to record any additional potential water contact sites.

#### Snail surveys

All water contact sites identified through the ethnographic observations were included in the snail surveys. We collected snails during four quarterly quantitative surveys over the period of 1 year, performed in March, July and October 2018 and February 2019 (Fig. 2), to assess potential differences in seasonality. Our aim was to complete two surveys per water contact site per time point; however no surveys were carried out in sites where water depth was too high to be safely entered at the time of the survey. Snail surveys followed the standard WHO protocol [33]: in brief, a handheld snail scoop was used to scoop the water body floor for 30 min per site and all scooped snails were collected. Physicochemical water factors, including temperature, total dissolved solids, pH and conductivity, were measured using a handheld water meter (model ProDSS; YSI Inc., Yellow Springs, OH, USA) at each site at each time point and recorded on a paper form. After collection, snails were sorted by genus based on shell morphology, counted and individually placed in wells of shedding plates in 3 ml bottled water to microscopically check for cercarial shedding.

After the initial check of the collected snails, non-shedding snails were housed in a well-ventilated aquaria at the Ugandan Ministry of Health—Vector Control Division, in Kampala, at room temperature, with frequent water changes, fed on dried lettuce ad libitum and monitored for 3 consecutive weeks to maximise the likelihood of detecting an infected snail, as cercariae can take up to several weeks to be released [34]. Snails were checked weekly for cercarial shedding by placing them under a warm indirect light source to induce cercarial release.

All snail collections, identification of snail genera and cercariae species, and monitoring for shedding were performed by trained staff members (FB and others) of the Ugandan Ministry of Health—Vector Control Division.

### Data analysis

#### Parasitological surveys

Data from the parasitological surveys were double entered into Microsoft Excel (Microsoft Corp., Redmond, WA, USA) and imported into R 4.0.4 [35] for merging, cleaning and analysis. The infection status of each participating child was determined at all sampling time points, and mean infection intensities were classified as per WHO guidelines, with a mean epg of 1–99 classified as low infection intensity, 100–399 epg as moderate and ≥ 400 epg as high infection intensity [36]. Medians and inter-quartile ranges (IQR) of infection intensities by risk group and sampling week were calculated and compared using Mann–Whitney U-tests.

#### Ethnographic data

Detailed notes from daily observations were transcribed, coded, categorised and compared using NVivo 12 [37] and Microsoft Excel.

#### Snail surveys

Survey data were double entered into Microsoft Excel and imported into R. Snail abundance, distribution of species and occurrence of cercarial shedding were calculated by water contact site and survey time point, and snail abundances were compared using Kruskal–Wallis tests and Mann–Whitney U-tests. Generalised linear mixed models (GLMM) using the R package *glmmtmb* [38] were performed to assess the effect of physicochemical water factors on snail abundance. Collinearity was assessed by the variance inflation factor score (VIF) using the package *car* [39]. Based on the differences in water site attendance and frequency of contact with water sites between CLI and CRI, as well as differences in high-risk behaviours, such as swimming, sites were assigned a low- or high-risk status. Maps were produced with QGIS 3.14 [40] using reference maps from Google Maps (satellite imagery) and Uganda Bureau of Statistics (district boundaries) [41].

## Results

### Cohort

Sixteen children were initially selected for participation in the study. The group of CLI comprised four girls and four boys, equally distributed by village, ranging in age from 8 to 14 years (median: 11 years). The group of CRI comprised five boys and three girls, ranging in age from 6 to 13 years (median: 10), with five children living in Bugoto A and three in Bugoto B. Representation of religions was similar in both groups: three CLI were Muslim, four CRI were Muslim; all others were Christian. In both groups, one child was not present at the time of the second set of ethnographic observations; both were replaced with children with similar characteristics, resulting in data collected from 18 children in total.

### Parasitological surveys

**Fig. 3:**
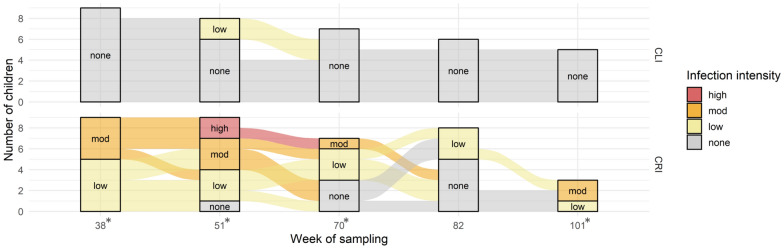
Infection status and intensity of selected children with rapid reinfection (CRI) and no or low-intensity infection (CLI) by sampling time point (weeks from first sample collection). An asterisk (*) by the week number indicates a significant result (*P* < 0.05, Mann–Whitney U-test) comparing CLI and CRI infection intensities

The post-selection parasitological surveys (weeks 51–101) showed significant and sustained differences in infection status and intensity between CRI and CLI over time (Fig. 3). For the majority of CRI, infections were detected at each time point, despite repeated praziquantel treatment. In contrast, no or few schistosome eggs were found in CLI across the time points (Additional file: Table S1). Significantly higher infection intensities (in epg) were found in CRI compared to CLI for week 38 (median [M] = 96 [IQR: 48–240] vs M = 0 [IQR: 0–0]; *P* < 0.001), week 51 (M = 105 [IQR: 45–144] vs M = 0 [IQR: 0–2]; *P* = 0.002), week 70 (M = 12 [IQR: 0–54] vs M = 0 [IQR: 0–0]; *P* = 0.031) and week 101 (M = 132 [IQR: 78–138] vs M = 0 [IQR: 0–0]; *P* = 0.017), but no difference was found for week 82 (M = 0 [IQR: 0–15] vs M = 0 [IQR: 0–0]; *P* = 0.127). Although not all children were present at every time point for the parasitological surveys (1 child was absent in week 51; 4 children were absent in weeks 51, 70 and 82; 10 children were absent in week 101), differences in infection status between the two groups are consistent throughout the observation period.

### Ethnographic observations

#### Water sources

There are several types of water sources in Bugoto, both natural and man-made. The largest permanent source of water is Lake Victoria, with adjacent swamps (Fig. 1). Inland of Bugoto B there are some rice paddies, ditches and ponds, some of which are temporary and can dry up in the dry season. Rainfall provides an additional source of water during the rainy seasons. At the time of the study, there were no piped water systems in Bugoto A or B; however the villages have a small number of boreholes with taps, some of which were out of order at the time of the study and some of which were in use, with a pay-per-quantity system. The nearest freely accessible borehole with a tap was a few kilometres outside of Bugoto in a different village. In addition to the distance, there were often long queues by the tap. Small plastic sachets of drinking water are also sold in the shops in Bugoto A and B.

#### Exposure behaviours

Children had skin contact with water for a wide range of activities (Table 1), at the sources directly as well as later on with water collected and then transported away from the collection source. Direct exposure was mainly through fetching water for the household, which 14 of the total 18 children recruited across both data collection periods carried out at least once during the observations. Children accessed the water barefoot, standing submerged at different depths of between the ankles and the hips for a few minutes at a time to fill the jerrycans, and subsequently exiting the water. We did not observe noticeable differences in the frequency and duration of household water fetching or in submersion depth between the CLI and CRI children. However, in comparison to CLI, CRI had additional water contact behaviours, in particular, fetching water for money and swimming in the lake which were not observed among any of the CLI. Four children engaged in swimming or playing in the water up to 4 times per day, ranging from 8 to 50 min of water contact in total. These children were all boys in the CRI group, aged up to 10 years and from both villages and religions. Three CRI, including one who also engaged in the swimming activity, collected water for other community members in return for money, up to five times per day. This group comprised both genders and religions and were of different ages, but all lived in Bugoto A, the village on the shores of the lake. The duration of this commercial fetching activity and the submersion depth were similar to those of household fetching, consisting of a few minutes each time and a depth ranging up to waist height. Only one child, a male CRI from Bugoto A, was observed to play with the collected water at home, pouring a layer of water from a jerrycan in a room with a concrete floor to use in a slip and slide activity.

**Table 1:** Observed water contact activities among participating children with rapid reinfection and no/low-intensity infection

Water contact activity	CRI	CLI
Bathing at home	Yes	Yes
Washing hands/feet/face	Yes	Yes
Washing clothes at home	Yes	Yes
Washing crockery at home	Yes	Yes
Washing crockery at school	Yes	Yes
Washing food items at home	Yes	Yes
Mopping house (bare hands/feet)	Yes	Yes
Playing with water at home	Yes	No
Fetching water for household purposes	Yes	Yes
Fetching water for money	Yes	No
Swimming at lake	Yes	No

*CLI* Children with no or low-intensity infection,* CRI* children with rapid reinfection

**Fig. 4:**
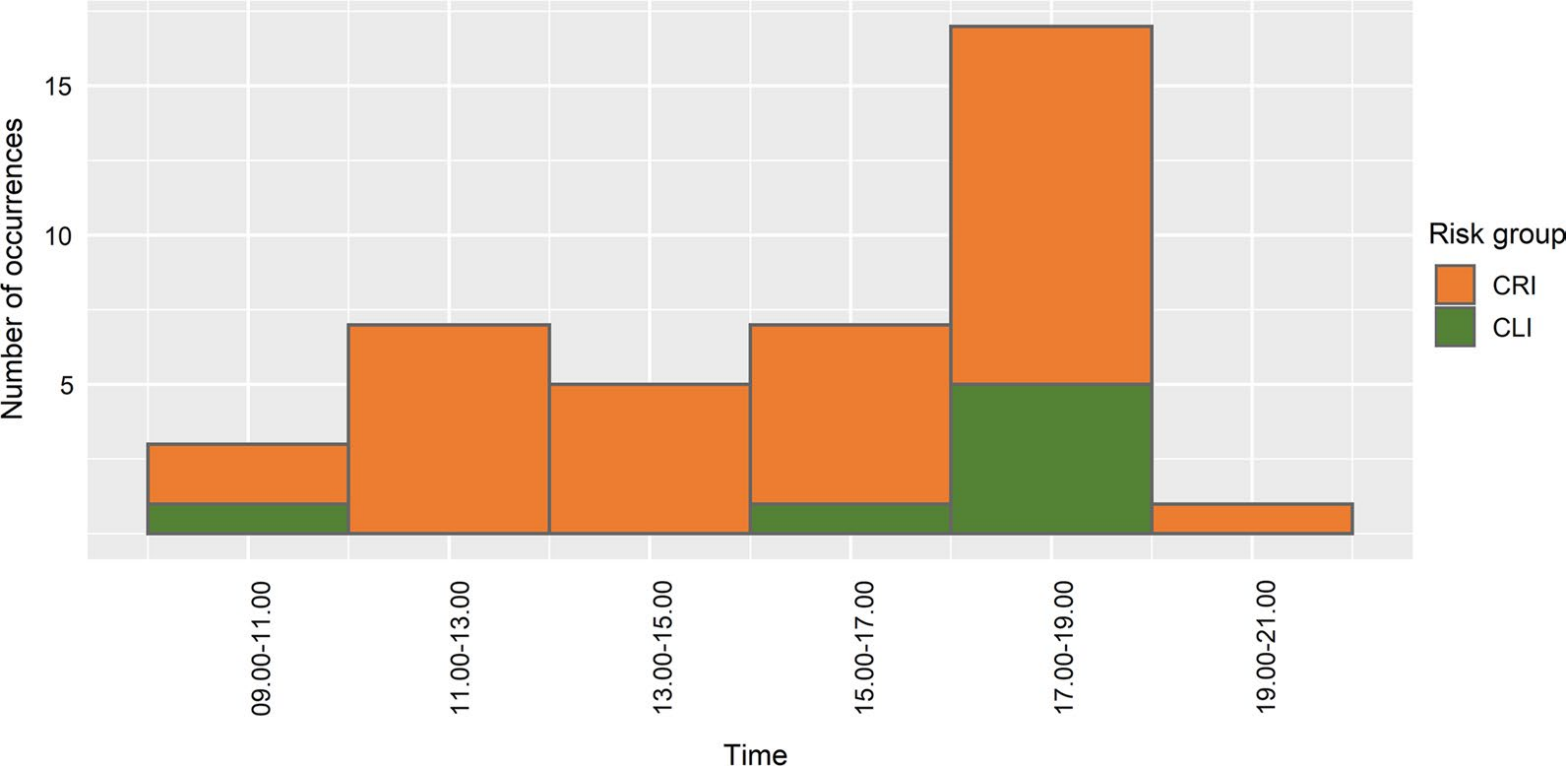
Observed direct water contact occurrences by time and risk group

Across all observation days, CLI accessed six different water contact sites and CRI accessed nine sites. Five CLI accessed one site, two accessed two water sites and two accessed three sites. CRI accessed a larger variety of sites than CLI, with one CRI accessing one site, three accessing two different sites, three accessing three different sites and two accessing four different sites. Both CLI and CRI had direct water contact most often between 1700 hours and 1900 hours (Fig. 4), with almost half of the total water contact episodes occurring during this time. Although we recorded only two instances of direct water contact among CLI before 1700 hours (one between 0900 hours and 1100 hours, and one between 1500 hours and 1700 hours), CRI accessed the water several times throughout the day, including around the midday; only one child accessed the water after 1900 hours, when it was already dark.

Children collected water in hard plastic jerrycans, mainly 20 l; however smaller jerrycans of 10 l and 5 l were also used, especially by young children who could not yet carry large jerrycans. Full jerrycans were mostly carried back by hand or on the head, but they were sometimes put on or tied to bicycles. In case of missing caps, children sometimes used thick leaves to prevent spillage.

The children placed the jerrycans with collected water by the house for immediate or later household use; however it was not recorded how long the water was stored before use. No households reported, or were observed, processing the water to eliminate potential pathogens before use. Some children heated bathing water for their parents on a fire, but this was reportedly done for warmth on cooler days, not for infection prevention.

Collected water was used for many purposes. Children used water for washing foods before cooking, for mopping the house floors (barefoot or in flipflops) and for washing their hands, feet or face. They added soap from soap bars to water for washing crockery and cutlery, washing jerrycans, washing clothes and footwear as well as for bathing and helping household members to bathe. We did not observe differences between CLI and CRI in the frequency or duration of these water contact activities in the household.

Half of the households collected rain water, an activity carried out more often by households of CLI (*n* = 6) than households of CRI (*n* = 3). People mainly collected rain by putting jerrycans out when it rained: one family had a larger open plastic tank next to their house and another family had constructed a round brick cistern on their compound. Rain water was used both for drinking and for household use. Other water for drinking was reported to come from boreholes, and no child or other household member was observed to drink water from lakes, swamps, ditches or ponds, either directly or after collection.

We carried out observations on an equal number of school days for CLI and CRI (both 9 school days vs 7 non-school days). Three children were found skipping a full day of school, all of whom were CRI. There was a higher frequency of all water contact activities on non-school days. Swimming and commercial fetching were only observed on non-school days. We observed more water contact on non-school days than on school days for several water contact activities; children fetched water for the household on 71% of non-school days vs 39% of school days, this was 36% and 6% for washing clothes respectively, 71% and 50% for washing plates and 86% and 72% for bathing. The only water contact children had at school was washing hands after eating a snack or lunch, reflected in a minor difference in handwashing between non-school days (79%) and school days (72%).

### Snail surveys

Through the ethnographic observations, 10 water contact sites were identified (A–J, Fig. 1). Most sites were by Lake Victoria (A–F), two were ponds (H, J), one was a swamp (G) and one was a ditch (I). Sites A–G were in the more densely populated area of Bugoto A; sites H, I and J were in rural areas of the surrounding villages. Site A was excluded from the survey in March, July and October and site B in July due to the water levels at these sites being too high to safely scoop for snails.

**Fig. 5:**
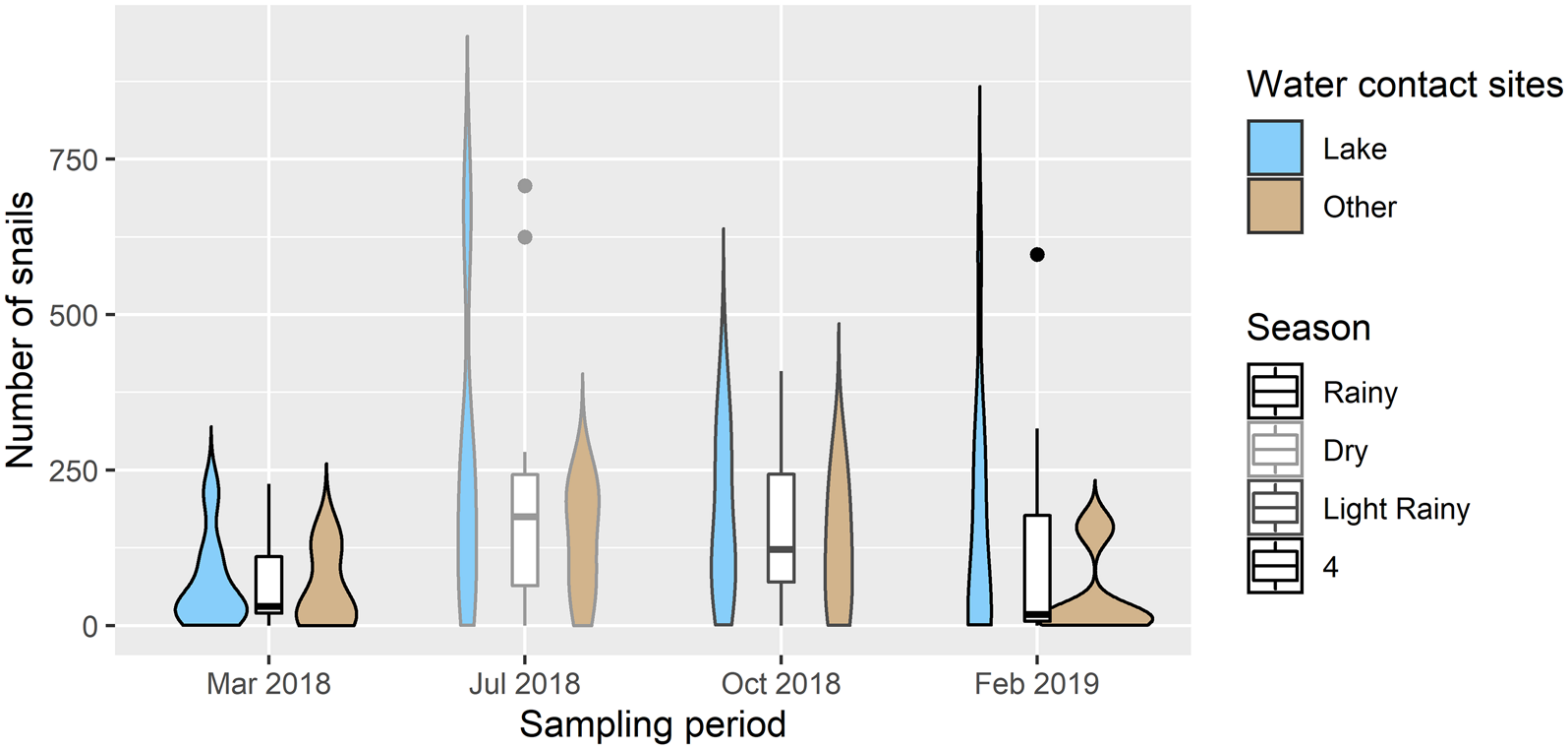
Abundance of *Biomphalaria* spp. by season and type of water contact site. Filled-in violin plots show the observed density within lake sites compared to non-lake sites (swamp, ditch, pond) for each sampling period. Boxplots show the interquartile range of all sites (lake and non-lake) by sampling period. Filled circles indicate outliers, and the line indicates median snail abundance per sampling unit. The outline shades of boxplots reflect the seasonal conditions (March 2018 and February 2019—rainy season; July 2018—dry season; October 2018—light rainy season)

In total, we collected 9457 *Biomphalaria* snails across the four time points and 10 sites (Fig. 5; Additional file 2: Table S2). There was a significant difference in median abundance across seasons (Kruskal–Wallis, *χ*^2^ = 10.496, *P* = 0.015). Highest abundance was found in July 2018, during the dry season (M = 175.50 [IQR: 64.25–242.50]), which was significantly higher than in the rainy seasons of March 2018 (M = 31.00 [IQR: 20.25–111.25]; *P* = 0.002) and February 2019 (M = 18.00 [IQR: 7.00–177.00]; *P* = 0.027) but not significantly higher than the light rainy season of October 2018 (M = 122.50 [IQR: 70.25–243.50]; *P* = 0.654). Slightly higher abundances of *Biomphalaria* snails were found in lake sites in comparison to other water bodies; however the difference was not significant (M = 92 [IQR: 27–228] vs M = 69 [IQR: 11–147]; *P* = 0.17). Site I was the only water contact site where no *Biomphalaria* snails were found during any of the surveys.

Of those collected snails whose species was identified (*n* = 6149), 64% were *Biomphalaria choanomphala*, 20% were *Biomphalaria sudanica* and 15% were *Biomphalaria pfeifferi* (Additional file: Table S2)*.* At the water contact sites by the lake (Fig. 1, sites A–F), the majority of snails were *B. choanomphala* (90%), whereas in the ponds, swamp and stream *B. sudanica* (58%) and *B. pfeifferi* (42%) were more frequently present and abundant*.*

**Fig. 6:**
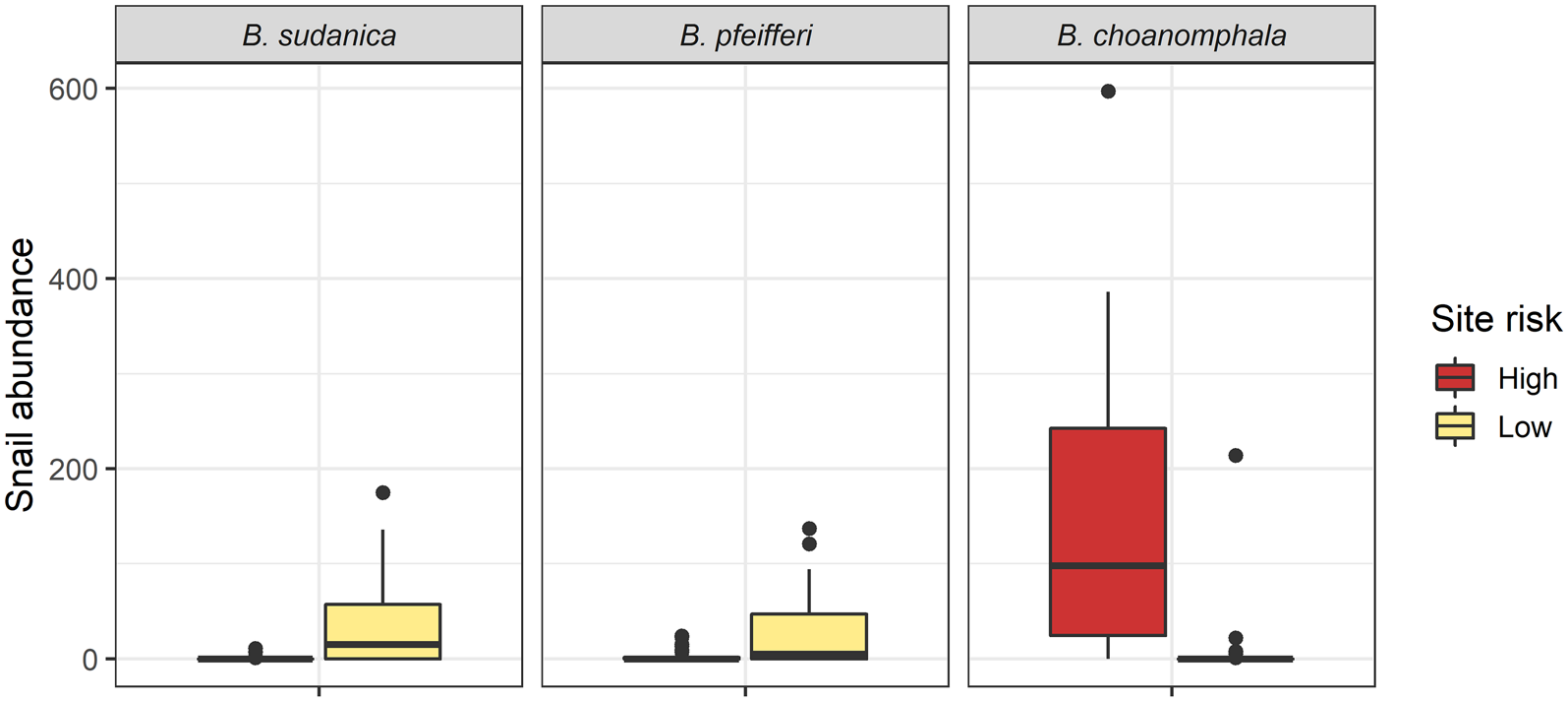
Snail abundance by site risk and species. Boxplots show the interquartile range of abundance in low-risk sites compared to high-risk sites by *Biomphalaria* species. Filled circles indicate outliers and the line indicates median snail abundance

Sites A, B, G, H, I and J were designated low-risk sites and sites C, D, E and F high-risk sites (Additional file 3: Table S3). High-risk sites were associated with a significantly higher abundance of *B. choanomphala* (M = 98 [IQR: 24.5–242.5] vs low-risk sites M = 0 [IQR: 0–0]; *P* < 0.001; Fig. 6) and low-risk sites with a significantly higher abundance of *B. sudanica* (M = 15 [IQR: 0–57] vs high-risk sites M = 0 [IQR: 0–0]; *P* < 0.001) and *B. pfeifferi* (M = 5 [IQR: 0–47] vs high-risk sites M = 0 [IQR: 0–1.5]; *P* = 0.003). Although physicochemical factors (temperature, pH, conductivity, total dissolved solids) varied between water contact sites (Additional file 4: Table S4; Additional file 5: Table S5), no variables were significantly different between the designated low- or high-risk sites.

Of the 9457 collected *Biomphalaria* snails, six snails (0.06%) shed *S. mansoni* cercariae during the 3 weeks of post-collection monitoring. Four shedding snails were *B. choanomphala* and two were *B. sudanica.* They were found during the March 2018 survey (*n* = 5) and in October 2018 (*n* = 1). Two were collected from site E, and one each from sites B, C, D and G. From the six snails that were found shedding cercariae, four were from high-risk sites.

## Discussion

Although in recent years progress has been made towards decreasing the burden of schistosomiasis in several endemic countries [42], areas with persistent high endemicity with rapid reinfections after treatment remain a major challenge for schistosomiasis control [43]. Despite very high prevalence in some of these communities, not everyone is infected, and understanding how individuals do not become infected in these areas may help to guide novel and current interventions. Here we demonstrate consistent and sustained differences in infection risk among SAC within an area with persistent high *S. mansoni* endemicity in Uganda. We integrated parasitological, ethnographic and malacological methods to identify water contact behaviours and locations, and environmental factors at these locations among SAC, and explored differences between children with rapid reinfection (CRI) and those with no/low-intensity infections (CLI). By using mixed methodologies, we were able to differentiate key risk behaviours and locations that have implications for differences in schistosomiasis infection within a persistent high endemic area.

SAC in this setting had frequent direct and indirect water contact for a range of purposes. The ethnographic observations highlighted several direct water contact risk behaviours by CRI that were not observed among CLI. Only CRI were observed to go swimming, an activity found to be a risk factor for schistosomiasis infection in previous studies among SAC [44, 45]. Swimming could contribute to a higher infection risk due to the greater degree of body submersion in the water and, therefore, increased skin contact with water, as well as the observed additional and extended time of water contact. Integrated schistosomiasis control through the development of safe water supplies, although important, does not affect the risk of infection from swimming. Swimming often has a different purpose, such as recreation [46] or cooling down from the heat [47]. In order to address this identified risk in CRI, additional interventions, such as parental guidance or community supervision, aimed at prohibiting children from going swimming [48, 49] may have an effect, but alternative options for cooling down or recreation [50] should be considered.

To our knowledge, this study is the first to report the practice of commercial water fetching; some children, all of them CRI, fetched water for other community members in return for money. This practice not only adds to the frequency of water contact but could also pose an economically compelling alternative to attending school, especially in an area where the mean daily income is around $1/day [51]. We observed more water contact during the days when children were not in school and some CRI were even skipping school days, suggesting school attendance may—aside from the educational benefits—reduce exposure and infection. In addition, where MDA is carried out in school settings, school non-attendance could mean some high-risk children may not be reached [52].

For most household chores involving water contact we did not observe considerable differences in the frequency and duration of water contact and in depth of submersion in water between the two groups. Collected water was not treated or processed (e.g. left in the sunshine) by both groups, and although we did not record how long water was stored before use, a practice that can decrease the number of viable cercariae in water [15], we learned from discussion with community members, Ugandan ethnographers working in this village and Ministry of Health technicians that water is rarely stored long enough for natural cercarial death. Further research on the storing of water at a household level could provide additional understanding around indirect exposure and possible differences in infection risk. For some chores, such as handwashing, bathing and washing clothes and dishes, infection risks could have also been minimised with the use of soap, which has been reported to kill cercariae [53, 54]. Although other possible protective measures, such as wearing gloves and boots, could be used for household chores with previously collected water, submersion when fetching water is often too deep for these measures to be useful at the water contact sites, and structural interventions such as jetties may be more beneficial.

Drinking water was retrieved from safe water sources, but the additional time, cost or effort this took was possibly too great for the larger water needs for household use. Households of CLI collected rain water more often than households of CRI. Until sufficient and accessible safe water is supplied, appropriate containers to collect rain could be used to increase safe water security in the rainy seasons.

Children from both groups had direct water contact most often in the after-school hours between 1700 hours and 1900 hours. CRI however were observed entering the water throughout the day, while no CLI were seen entering the water at midday, when cercarial shedding is thought to be the highest [55, 56]. Timing of direct water contact could therefore pose an additional risk for CRI. Children with rapid reinfection also accessed a larger variety of water contact sites than CLI. Multiple water source use is common in low- and middle-income countries, with the reasons including seasonality, perception of quality, distance or cost [13]. This practice could possibly impact infection risk in areas where infection risk between water contact sites is not homogeneous, such as in our study. Sites annotated as high-risk sites, combining observed risk behaviour and site attendance, were mainly lake sites with high human activity and nearby latrines. These sites had a higher abundance of *B. choanomphala* snails in comparison to the low-risk, mostly non-lake, sites where *B. pfeifferi* and *B. sudanica* were mainly found. These results are in line with those of previous studies where these latter species were also mostly found in shallow swampy waters [25, 28, 57]. The WHO urges snail control to become a more prominent part of control strategies [58], including in areas with persistent high endemicity [33]. Although the impact of chemical-based molluscicides on schistosomiasis transmission has been reported in several studies, challenges include cost, toxicity and the need for regular application [59]. We recorded 10 water contact sites for the children in our study, adding to the complexity of vector control required in this setting. Novel ecological solutions, such as introducing snail predators, could offer a lower cost, more sustainable option [60]. In this setting, the sheer volume of Lake Victoria poses additional complexities and, therefore, treating the smaller non-lake water bodies would be more manageable; however our findings suggest this would mainly target already lower-risk sites and low-risk children.

A larger proportion of snails infected with *S. mansoni* cercariae were *B. choanomphala*. This species has been found to be more susceptible to *S. mansoni* than *B. pfeifferi* [22] or *B. sudanica* [61], but snail infection rates in our study were too low overall (0.06%) to explicitly show differences in susceptibility among snails in this community. Finding low numbers of snails shedding cercariae in an area with high prevalence of human infections has been reported in several settings [23, 24, 62]. Although the shedding method could underestimate the infections in snails [63], even a low prevalence of snails releasing cercariae may be sufficient to sustain transmission, if exposure is frequent and prolonged, such as found in this study. Additionally, we found very large populations of snails, indicating that even a very low infection prevalence would still result in high numbers of infected snails overall, especially as many of these sites were perennial snail sites capable of supporting year-round transmission.

In the study area, temperatures are also suitable for snail and parasite survival throughout the year. Temperature was positively associated with snail abundance, and the highest abundance of snails was found in the dry season, similar to results from other studies carried out around Lake Victoria [20, 22]. Rainfall is suggested to increase pH and turbidity [20], which were also negatively associated with snail abundance in our study. Although snails were found all year around and, therefore, infection potential is continuously present, the occurrence of more snails during the dry season is of concern. Although water contact behaviour during the dry season was not recorded in this study, children have been observed to swim more during the dry season [49, 64, 65], as it is the hottest season and swimming in water is refreshing. [66]. In addition, during the dry season, rain water is not available and some smaller water bodies dry up, possibly making people divert to permanent water bodies, as observed in Kenya [67] and Senegal [68], which in this study area is associated with a higher risk of water contact. Additional research on water contact behaviours across seasons could therefore provide greater insights for this. Furthermore, if exposures are highest in the dry seasons, MDA may, therefore, be most effective if planned directly after the dry season, although further research would be needed to confirm this.

Our in-depth focus on these two villages provided the opportunity to combine several research methods to gain a more complete understanding of variations in infection risk. The study community is comparable to other communities in the district [51] and diverse in aspects such as density, livelihood and distance to the water bodies. Even given the limited geography of the study area and the limited size of the study population, we found significant and sustained differences in infection status and infection intensity between the two risk groups. No heavy infections were found after repeated treatments with praziquantel, an encouraging finding towards the target of < 1% heavy infections by 2030 expressed in the recently published Roadmap for Neglected Tropical Diseases [1]. Although water contact sites were identified based on ethnographic observations of the selected CRI and CLI, regular presence in the community of the study team members, transect walks through the area and discussion with people from the community did not reveal any major water contact sites that possibly had been missed, indicating that these findings are relevant for the wider community.

While we used a standardised method for snail collection, deep water contact sites could not be sampled safely. Water depth in these sites were seasonal and limited our access, but water contact by study participants and other community members still occurred. Future research could consider conducting snail collection at these sites with longer scoops or other methods, such as dredging [69], or taking water samples instead to detect cercariae using fluorescent assays [70]. In addition, citizen science projects provide an opportunity to increase the frequency of snail collection to more consistently monitor snail species abundance and distribution as well as increase awareness and participation among the communities affected by schistosomiasis [71].

Another limitation of the study could have been that the presence of the researchers, both long-term in this village and during the ethnographic data collection, may have changed the behaviours of the children being studied. However, this was minimised by having a community member in the research team during the ethnographic work who was aware of any atypical behaviour that may have been biased, and this topic was openly discussed. Furthermore, there is no reason to believe that the children, who did not know they were classified into the two CLI and CRI groups, would have changed their behaviour differently, indicating that these findings are likely representative of these two groups of SAC. Findings from this study also provided input into ongoing qualitative research with SAC and their parents to further understand perceptions and attitudes towards water contact and schistosomiasis more generally.

## Conclusions

The findings of this study highlight specific water contact behaviours and environmental risk factors that can explain variation in *S. mansoni* infection risk in SAC within an area with persistent high endemicity. We recommend complementing existing MDA programmes with targeted vector control, safe water supply, including collection of rain water, as well as addressing directly contributing factors, such as commercial water fetching and swimming, and indirect factors, such as increased school attendance, in order to reduce (re)infections in these highly endemic settings.

## Supplementary Information

**Additional file 1: Table S1.** *Schistosoma mansoni* infection status and intensity (mean number of eggs per gram of stool from 1–3 days of duplicate kato-katz thick smears) per timepoint for CLI and CRI. Week 0 occurred in March 2017.

**Additional file 2: Table S2.** Number of collected *Biomphalaria* snails by data collection time point, water contact site (A–J) and species. Abbreviations:* Bs*, *Biomphalaria sudanica*;* Bp*, *Biomphalaria pfeifferi*;* Bc*, *Biomphalaria choanomphala*).

**Additional file 3: Table S3.** Infection risk factors and site risk classification.

**Additional file 4: Table S4** Temperature, pH, total dissolved solids and conductivity by type of water contact site. Temperature was not found to be different between lake and non-lake sites, but pH was significantly higher for the lake sites. Total dissolved solids and conductivity were significantly higher in the non-lake sites.

**Additional file 5: Table S5.** Best-fitted generalised linear mixed models (GLMM) of *Biomphalaria* spp. abundance and physicochemical factors by type of water contact site. Temperature showed a significantly positive association with snail abundance in lake sites as well as non-lake sites. Increased pH had a significantly negative association with abundance in both lake sites and non-lake sites and a slight negative association was also found with conductivity.

## Data Availability

The datasets from snail surveys generated and analysed during the current study are available in the Enlighten repository of the University of Glasgow (10.5525/gla.researchdata.1233). All parasitological data generated or analysed during this study are included in this published article (and its additional information files). The data from ethnographic observations generated and analysed during the current study are not publicly available due to privacy reasons but are available anonymised where possible from the corresponding author on reasonable request.

## Abbreviations

CLI: Children with no or low-intensity infection and no infection after clearance
CRI: Children with rapid reinfection
epg: Eggs per gram
GLMM: Generalised linear mixed models
MDA: Mass drug administration
SAC: School-aged children
VIF: Variance inflation factor score
WHO: World Health Organization
