# Supplementary material for: Variation in water contact behaviour and risk of Schistosoma mansoni (re)infection among Ugandan school-aged children in an area with persistent high endemicity
Source: Parasit Vectors. 2022 Jan 6;15:15. doi: 10.1186/s13071-021-05121-6 (PMC8734346; doi:10.1186/s13071-021-05121-6)
Supplement: Supplementary file 1 — Additional file 1: Table S1. Schistosoma mansoni infection status and intensity (mean number of eggs per gram of stool from 1–3 days of duplicate kato-katz thick smears) per timepoint for CLI and CRI. Week 0 occurred in March 2017. [file 13071_2021_5121_MOESM1_ESM.docx]

|  | **Week since first sample collection** | | | | | | | |
| --- | --- | --- | --- | --- | --- | --- | --- | --- |
|  | **0** | **28** | **32** | **38** | **51** | **70** | **82** | **101** |
| **CLI ID** | Mean epg | | | | | | | |
| 1 | 0 | 4 | 0 | 0 | 8 | 0 | 0 | 0 |
| 2^b^ | 0 | 0 | 0 | 0 | 0 | 0 | na | na |
| 3 | 0 | 0 | 0 | 0 | 0 | 0 | 0 | 0 |
| 4^c^ | 28 | 0 | 0 | 0 | 0 | na | 0 | 0 |
| 5 | 0 | 4 | 0 | 0 | 0 | 0 | na | na |
| 6 | 12 | 0 | 0 | 0 | 0 | 0 | 0 | 0 |
| 7 | 0 | 0 | 0 | 0 | 12 | 0 | 0 | 0 |
| 8 | 4 | 0 | 0 | 0 | 0 | na | na | na |
| 9 | 8 | 0 | 0 | 0 | na | 0 | 0 | na |
| **CRI ID** |  | | | | | | | |
| 10 | 628 | 20 | 0 | 372 | 15 | 36 | 0 | 144 |
| 11 | 0 | 40 | 0 | 96 | 141 | 12 | 0 | na |
| 12 | 712 | 160 | 0 | 36 | 60 | 72 | 12 | na |
| 13 | 3120 | 1896 | 0 | 132 | 480 | 288 | 0 | na |
| 14 | 619 | 48 | 0 | 240 | 144 | 0 | 24 | 132 |
| 15 | 76 | 32 | 0 | 66 | 105 | 0 | 0 | 24 |
| 16 | 2616 | 1380 | 0 | 48 | 45 | 0 | 48 | na |
| 17^c^ | 1108 | 16 | 0 | 12 | 0 | na | 0 | na |
| 18^b^ | 68 | 0 | 0 | 276 | 474 | na | na | na |

^b^ Replacement participants, only included in October observations

^c^ Participants only included in March observations, lost to follow-up
