# Supplementary material for: Variation in water contact behaviour and risk of Schistosoma mansoni (re)infection among Ugandan school-aged children in an area with persistent high endemicity
Source: Parasit Vectors. 2022 Jan 6;15:15. doi: 10.1186/s13071-021-05121-6 (PMC8734346; doi:10.1186/s13071-021-05121-6)
Supplement: Supplementary file 2 — Additional file 2: Table S2. Number of collected Biomphalaria snails by data collection time point, water contact site (A–J) and species. Abbreviations: Bs, Biomphalaria sudanica; Bp, Biomphalaria pfeifferi; Bc, Biomphalaria choanomphala). [file 13071_2021_5121_MOESM2_ESM.docx]

|  | **March 2018** | | | | **July 2018** | **October 2018** | | | **February 2019** | | | | **Total** | | | | |
| --- | --- | --- | --- | --- | --- | --- | --- | --- | --- | --- | --- | --- | --- | --- | --- | --- | --- |
| **Site** | *Bs*  (n) | *Bp*  (n) | *Bc*  (n) | *B. spp.*  (n) | | *Bs*  (n) | *Bp*  (n) | *Bc*  (n) | | *Bs*  (n) | *Bp*  (n) | *Bc*  (n) | | *Bs*  (n) | *Bp*  (n) | *Bc*  (n) | *B. spp.*  (n) |
| **A** | - | - | - | - | | - | - | - | | 0 | 7 | 9 | | 0 | 7 | 9 | **16** |
| **B** | 65 | 7 | 23 | - | | 141 | 117 | 0 | | 0 | 5 | 13 | | 206 | 129 | 36 | **371** |
| **C** | 0 | 1 | 20 | 400 | | 0 | 15 | 237 | | 0 | 0 | 0 | | 0 | 16 | 257 | **673** |
| **D** | 1 | 2 | 168 | 1332 | | 0 | 9 | 110 | | 0 | 0 | 291 | | 1 | 11 | 569 | **1913** |
| **E** | 0 | 0 | 549 | 98 | | 0 | 36 | 689 | | 0 | 0 | 795 | | 0 | 36 | 2033 | **2167** |
| **F** | 18 | 0 | 91 | 529 | | 0 | 31 | 353 | | 0 | 0 | 615 | | 18 | 31 | 1059 | **1637** |
| **G** | 52 | 1 | 0 | 444 | | 305 | 215 | 0 | | 20 | 28 | 0 | | 377 | 244 | 0 | **1065** |
| **H** | 258 | 10 | 1 | 363 | | 115 | 141 | 0 | | 101 | 216 | 0 | | 474 | 367 | 1 | **1205** |
| **I** | 0 | 0 | 0 | 0 | | 0 | 0 | 0 | | 0 | 0 | 0 | | 0 | 0 | 0 | **0** |
| **J** | 15 | 0 | 0 | 142 | | 129 | 104 | 0 | | 10 | 10 | 0 | | 154 | 114 | 0 | **410** |
| **All** | **409** | **21** | **852** | **3308** | | **690** | **668** | **1389** | | **131** | **266** | **1723** | | **1230** | **955** | **3964** | **9457** |
