# Supplementary material for: Variation in water contact behaviour and risk of Schistosoma mansoni (re)infection among Ugandan school-aged children in an area with persistent high endemicity
Source: Parasit Vectors. 2022 Jan 6;15:15. doi: 10.1186/s13071-021-05121-6 (PMC8734346; doi:10.1186/s13071-021-05121-6)
Supplement: Supplementary file 3 — Additional file 3: Table S3. Infection risk factors and site risk classification. [file 13071_2021_5121_MOESM3_ESM.docx]

| **Site** | Water contact CRI | Water contact CLI | Swimming | Commercial water fetching | **Site risk** |
| --- | --- | --- | --- | --- | --- |
| **A** | + | + | - | - | **Low** |
| **B** | ++ | ++ | - | - | **Low** |
| **C** | +++ | +++ | + | - | **High** |
| **D** | ++ | + | + | + | **High** |
| **E** | ++ | - | + | + | **High** |
| **F** | + | - | - | + | **High** |
| **G** | + | - | - | - | **Low** |
| **H** | - | + | - | - | **Low** |
| **I** | + | - | - | - | **Low** |
| **J** | + | + | - | - | **Low** |
