# Supplementary material for: Variation in water contact behaviour and risk of Schistosoma mansoni (re)infection among Ugandan school-aged children in an area with persistent high endemicity
Source: Parasit Vectors. 2022 Jan 6;15:15. doi: 10.1186/s13071-021-05121-6 (PMC8734346; doi:10.1186/s13071-021-05121-6)
Supplement: Supplementary file 4 — Additional file 4: Table S4 Temperature, pH, total dissolved solids and conductivity by type of water contact site. Temperature was not found to be different between lake and non-lake sites, but pH was significantly higher for the lake sites. Total dissolved solids and conductivity were significantly higher in the non-lake sites. [file 13071_2021_5121_MOESM4_ESM.docx]

| **Physicochemical water factor** | **Water contact sites** | **Median** | **Inter-quartile range** | **Mann-Whitney W** | **p-value** |
| --- | --- | --- | --- | --- | --- |
| Temperature | Lake sites | 27.7 | [26.3-29.0] | 441 | 0.268 |
|  | Non-lake sites | 26.4 | [24.6-29.7] |  |  |
| pH | Lake sites | 8.1 | [7.8-8.5] | 540 | <0.001 |
|  | Non-lake sites | 7.2 | [6.9-7.3] |  |  |
| Total dissolved solids | Lake sites | 72 | [52-74] | 19 | <0.001 |
|  | Non-lake sites | 549 | [248-772] |  |  |
| Conductivity | Lake sites | 117 | [105-123] | 16 | <0.001 |
|  | Non-lake sites | 966 | [479-1389] |  |  |
