# Supplementary material for: Variation in water contact behaviour and risk of Schistosoma mansoni (re)infection among Ugandan school-aged children in an area with persistent high endemicity
Source: Parasit Vectors. 2022 Jan 6;15:15. doi: 10.1186/s13071-021-05121-6 (PMC8734346; doi:10.1186/s13071-021-05121-6)
Supplement: Supplementary file 5 — Additional file 5: Table S5. Best-fitted generalised linear mixed models (GLMM) of Biomphalaria spp. abundance and physicochemical factors by type of water contact site. Temperature showed a significantly positive association with snail abundance in lake sites as well as non-lake sites. Increased pH had a significantly negative association with abundance in both lake sites and non-lake sites and a slight negative association was also found with conductivity. [file 13071_2021_5121_MOESM5_ESM.docx]

| **Lake sites** | **Fixed effects** | **Estimate** | **Standard error** | **p-value** |
| --- | --- | --- | --- | --- |
|  | Intercept | 19.34 | 2.981 | <0.001 |
|  | pH | -0.97 | 0.063 | <0.001 |
|  | Conductivity | -0.06 | 0.003 | <0.001 |
|  | Temperature | 0.11 | 0.025 | <0.001 |
|  |  |  |  |  |
|  | **Random effects** | **Variance** | **Standard deviation** |  |
|  | Sampling period | 23.70 | 4.868 |  |
|  | Site | 2.52 | 1.587 |  |
| **Non-lake sites** | **Fixed effects** | **Estimate** | **Standard error** | **p-value** |
|  | Intercept | 9.19 | 1.863 | <0.001 |
|  | pH | -0.86 | 0.089 | <0.001 |
|  | Conductivity | -0.001 | 0.00002 | <0.001 |
|  | Temperature | 0.05 | 0.022 | 0.015 |
|  |  |  |  |  |
|  | **Random effects** | **Variance** | **Standard deviation** |  |
|  | Sampling period | 2.79 | 1.670 |  |
|  | Site | 6.18 | 2.486 |  |

Footnote: Total dissolved solids and conductivity were highly collinear (VIF 42 and 41 respectively), therefore total dissolved solids was not included in the model.

Footnote: As conductivity and pH both interacted with the type of site, separate GLMMs were fitted for lake sites and non-lake sites
